# Influence of Sodium Citrate Supplementation after Dehydrating Exercise on Responses of Stress Hormones to Subsequent Endurance Cycling Time-Trial in the Heat

**DOI:** 10.3390/medicina55040103

**Published:** 2019-04-12

**Authors:** Silva Suvi, Martin Mooses, Saima Timpmann, Luule Medijainen, Eve Unt, Vahur Ööpik

**Affiliations:** 1 Institute of Sport Sciences and Physiotherapy, University of Tartu, 50090 Tartu, Estonia; silva.suvi@ut.ee (S.S.); martin.mooses@ut.ee (M.M.); saima.timpmann@ut.ee (S.T.); luule.medijainen@ut.ee (L.M.); 2 Estonian Centre of Behavioral and Health Sciences, University of Tartu, 50090 Tartu, Estonia; 3 Department of Cardiology, University of Tartu, 50090 Tartu, Estonia; eve.unt@kliinikum.ee; 4 Department of Sports Medicine and Rehabilitation, Institute of Clinical Medicine, University of Tartu, 50090 Tartu, Estonia; 5 Sports Medicine and Rehabilitation Clinic, Tartu University Hospital, 50406 Tartu, Estonia

**Keywords:** aldosterone, prolactin, growth hormone, cortisol, plasma volume, exercise-heat stress

## Abstract

*Background and objectives:* In temperate environments, acute orally induced metabolic alkalosis alleviates exercise stress, as reflected in attenuated stress hormone responses to relatively short-duration exercise bouts. However, it is unknown whether the same phenomenon occurs during prolonged exercise in the heat. This study was undertaken with aim to test the hypothesis that ingestion of an alkalizing substance (sodium citrate; CIT) after dehydrating exercise would decrease blood levels of stress hormones during subsequent 40 km cycling time-trial (TT) in the heat. *Materials and Methods:* Male non-heat-acclimated athletes (*n* = 20) lost 4% of body mass by exercising in the heat. Then, during a 16 h recovery period prior to TT in a warm environment (32 °C), participants ate the prescribed food and ingested CIT (600 mg·kg^−1^) or placebo (PLC) in a double-blind, randomized, crossover manner with 7 days between the two trials. Blood aldosterone, cortisol, prolactin and growth hormone concentrations were measured before and after TT. *Results:* Total work performed during TT was similar in the two trials (*p* = 0.716). In CIT compared to PLC trial, lower levels of aldosterone occurred before (72%) and after (39%) TT (*p* ˂ 0.001), and acute response of aldosterone to TT was blunted (29%, *p ˂* 0.001). Lower cortisol levels in CIT than in PLC trial occurred before (13%, *p* = 0.039) and after TT (14%, *p* = 0.001), but there were no between-trial differences in the acute responses of cortisol, prolactin or growth hormone to TT, or in concentrations of prolactin and growth hormone before or after TT (in all cases *p* > 0.05). *Conclusions:* Reduced aldosterone and cortisol levels after TT and blunted acute response of aldosterone to TT indicate that CIT ingestion during recovery after dehydrating exercise may alleviate stress during the next hard endurance cycling bout in the heat.

## 1. Introduction

Ingestion of alkalizing substances like sodium bicarbonate (NaHCO_3_) or sodium citrate (CIT) may improve performance in high-intensity exercise [1,2,3]. The ergogenic effects of these substances are considered to be based mainly on their property of increasing extracellular buffer capacity, which facilitates efflux of hydrogen ions (H^+^) from contracting muscle cells [4,5,6], thereby delaying the fall in intracellular pH and enhancing glycogenolytic ATP production [7,8]. In addition, CIT has been shown to increase plasma volume (PV) [9,10] to an extent that may improve endurance performance through slowing down increases in core body temperature (T_c_) during exercise [11].

The majority of previous studies have focused on potential performance effects of acute ingestion of alkalizers, whereas their impact on exercise-induced stress has received little attention. McEwen [12] defined stress as “a real or interpreted threat to the physiological or psychological integrity (i.e., homeostasis) of an individual that results in physiological and/or behavioral responses”. Accordingly, the term stressor refers to any factor that causes stress, i.e., induces physiological and/or behavioral responses. Physiological responses to stress generally comprise changes in neuroendocrine, hormonal and immune functions [13,14]. Acute exercise threats homeostasis and depending on its intensity and duration, may be a powerful stressor [15,16]. Recent findings suggest that NaHCO_3_ alleviates stress at the cellular level, as revealed by attenuated responses of heat shock proteins to exercise [17,18]. Data on the responses of stress hormones to exercise after CIT or NaHCO_3_ ingestion is scarce and partially controversial. Attenuated [19] or unchanged [20,21] blood catecholamine responses to high-intensity cycling exercise of 1–2 min duration have been reported. NaHCO_3_ attenuated cortisol (CORT) and growth hormone (GH) responses to maximal cycling bouts [22,23]. NaHCO_3_ infusion blocked prolactin (PRL) response to approximately 7.5 min exhausting cycling bout [24]. CIT blunted aldosterone (ALDO) response to approximately 24.5 min incremental running exercise [10].

Thus, the majority of the available data suggest that acute orally induced metabolic alkalosis alleviates exercise stress, as reflected in attenuated stress hormone responses to exercise [10,19,22,23,24], but all of these studies were carried out in temperate environmental conditions and investigated relatively short-duration exercise bouts. However, unusually high environmental temperature is also a stressor [25,26]. Simultaneous influence of various stressors may provide a larger physiological stress to the body and induce larger neuroendocrine responses than exposure to a single stressor [27]. Indeed, prolonged exercise in the heat compared to the same exercise performed in a temperate environment induces greater increases in blood PRL [28,29], GH [30,31], CORT [32,33] and ALDO [34] levels, but to the best of our knowledge, it is unknown whether CIT or NaHCO_3_ ingestion alleviates responses of stress hormones to prolonged endurance exercise in warm environments.

Generally, stress induced by exercise is considered to be a stimulus for positive physiological adaptations that, in the course of systematic training lasting several weeks, months or years, leads to improvement of health and performance [15]. Nevertheless, the actual outcome of stress imposed by exercise may be positive or negative, depending on the athlete’s ability to adapt to the exercise load [15,27]. Important factors influencing the individual’s ability to adapt are the quality and quantity of recovery between training sessions. Therefore, after a relatively more stressful exercise, a relatively longer recovery period may be needed [27]. However, athletes often experience situations where time to recover is limited. Many multi-stage road cycling competitions, for example, take place in a warm climate. As general capacity for adaptation is finite [27], otherwise well-tolerable exercise leads may become less tolerable or even intolerable in conditions of cumulative exercise and heat stress. Considering that resting periods between consecutive stages are rather short, the quality of recovery becomes an especially important factor in determining the ability of an athlete to maintain high level performance over several days or even weeks of a competition. A recent publication [35] revealed that CIT ingestion after dehydrating exercise (DE) effectively stimulated rehydration, but still did not enhance performance during subsequent heavy exercise bout in the heat. In this paper, which is based on unpublished data collected in the same experimental study [35], we focus on the potential impact of CIT ingestion on the levels of stress hormones. By increasing the body’s buffer capacity [6,9] and PV [9,10], CIT may facilitate maintenance of homeostasis, i.e., reduce physiological stress, during heavy exercise in the heat.

Thus, our purpose was to test the hypothesis that CIT ingestion during 16 h recovery period after DE, in an amount that has been shown to induce metabolic alkalosis and expansion of PV, would attenuate increases in the blood levels of CORT, ALDO, GH and PRL during subsequent 40 km cycling time-trial (TT) in the heat. We experimented with CIT because this substance has been shown to cause less gastrointestinal distress than NaHCO_3_ [36].

## 2. Materials and Methods

### 2.1. Participants

A detailed description of the study protocol, participants, research procedures and apparatuses used is given in our previous publication [35]. Briefly, the study was approved by the Research Ethics Committee of the University of Tartu (protocol 244/T-16, 19 January 2015) and it was carried out in two periods in 2015: from February to April and from October to December. Prior to inclusion into the study, written informed consent was obtained from 20 non-heat-acclimated endurance-trained men (mean ± SD; age 30.8 ± 5.4 years, height 1.81 ± 0.07 m, body mass (BM) 78.2 ± 8.4 kg, peak oxygen uptake (VO_2_peak) 57.0 ± 5.9 mL·kg^−1^·min^−1^) [35].

### 2.2. Study Design and Research Procedures

The study was carried out in two stages: preparatory and main phase [35]. In the preparatory phase, during their first visit to the laboratory, participants’ anthropometric data was collected. In addition, their VO_2_peak was measured in temperate environmental conditions (21–22 °C, relative humidity (RH) 50–51%), employing test protocol and equipment described in detail by Suvi et al. [35]. During their next two visits to the laboratory, on both occasions, the participants performed a familiarization TT in the heat (32 °C; RH 46%).

**Figure 1:**
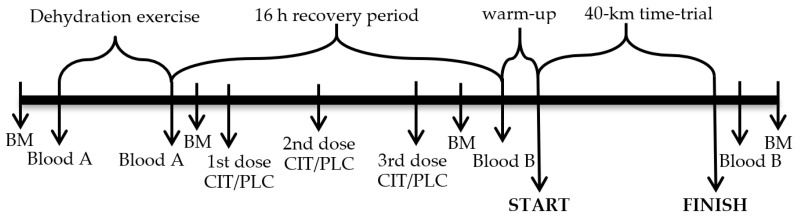
Study protocol. Arrows indicate time points for measurements or blood sampling, sodium citrate or placebo (CIT/PLC) administration and 40-km time-trial start and finish. Blood A indicates blood samples in which hematocrit and hemoglobin concentration was measured; Blood B indicates blood samples in which hematocrit and concentrations of hemoglobin, hormones, glycose and lactate were measured; BM is body mass.

Participants were instructed to refrain from strenuous exercise and to record their dietary intake 24 h before the main phase to ensure stable nutritional status. On the first day of the main phase (Figure 1), participants arrived at the laboratory at 2:30 PM. They voided, their nude BM was measured, and then, wearing only shorts and sport shoes, they entered the climatic chamber, where air temperature and RH were kept at 32 °C and 46%, respectively [35]. Participants remained in a sitting position for 20 min prior to donating a blood sample and starting DE. During DE, participants exercised on a cycle ergometer at 50–60% of their individual VO_2_peak until approximately 4% BM loss was achieved due to sweating [35].

A recovery period lasting for approximately 16 h started immediately after DE. During the recovery period, participants drank bottled water ad libitum, ate prescribed dinner (20 kcal·kg^−1^) and breakfast (12 kcal·kg^−1^), both in the laboratory and ingested gelatine capsules containing CIT (600 mg·kg^−1^) or PLC (sucrose) in a randomized, double-blind, crossover manner with 7 days between the two trials [35]. The first dose (200 mg·kg^−1^ initial BM) of CIT or PLC was administered during dinner (approximately 16 h prior to TT), the second dose (200 mg·kg^−1^) was ingested by participants roughly 1 h before bedtime (approximately 11 h prior to TT) at home, and the third 200 mg·kg^−1^ dose was consumed the next morning during breakfast at the laboratory at 8:00 AM (2 h prior to TT). The same total dose and identical schedule of CIT ingestion has been demonstrated to expand PV and significantly increase blood pH (from 7.40–7.44), bicarbonate ion concentration (from 25.0–30.45 mmol·L^−1^) and base excess (from 0.54–5.74 mmol·L^−1^) [9].

Participants’ nude BM was recorded approximately 60 min after the breakfast. After positioning a rectal temperature probe, participants entered the climatic chamber (32 °C; RH 46%) and spent 20 min in a sitting position prior to donating venous and capillary blood samples. Before starting the TT, participants slightly warmed up by cycling 5 min on a cycle ergometer. During the TT, participants had free access to water, their T_c_ was recorded every 1 min, and they were verbally encouraged to complete the exercise task as fast as possible [35].

### 2.4. Blood Sampling and Analyses

Venous blood samples were drawn at the antecubital fossa of the arm into Vacutainer tubes containing EDTA, and into sterile serum Vacutainer tubes shortly before and after DE and TT. In blood taken from the EDTA tube, hematocrit and hemoglobin concentration were measured [35], and on the basis of these data, changes in PV were calculated [37]. After keeping the remaining blood in EDTA and sterile serum tubes at room temperature for 10 min, the tubes were centrifuged for 10 min at 3000 rpm at 4 °C (Eppendorf 5804R, Eppendoff AG, Hamburg, Germany). Serum and plasma specimens were maintained at −25 °C until assayed for hormones and metabolites.

ALDO and GH concentrations were measured in plasma and serum, respectively, with chemiluminescence immunoassay method and using IDS-iSYS Multi-Discipline Automated Analyser (Immunodiagnostic Systems Limited, Boldon Colliery, UK). According to the manufacturer, the minimal detection limits of the assays were 3.7 ng·dL^−1^ and 0.15 mU·L^−1^, respectively. The intra- and inter-assay coefficients of variation (CV) were 1.7–8.4% and 5.2–12.8% for ALDO, and 1.8–3.5% and 5.9–10.4% for GH.

Concentrations of PRL and CORT were measured in serum using electrochemiluminescence immunoassay “ECLIA” on Cobas 6000 e601 analyzer (Roche Diagnostics GmbH, Mannheim, Germany). The lower detection limits of the assays were 1.00 mU·L^−1^ and 1.5 nmol·L^−1^, respectively. The intra- and inter-assay CVs were 0.8–1.7% and 1.4–2.0% for PRL, and 1.5–2.4% and 1.9–2.8% for CORT.

Glucose concentration in serum was measured using the hexokinase enzymatic method on Roche/Hitachi Cobas 6000 c501 analyser (Roche Diagnostics GmbH, Mannheim, Germany). Minimal detection limit, intra- and inter-assay CVs were 0.11 mmol·L^−1^, 0.7–0.8% and 1.2–1.4%, respectively.

Lactate concentration in capillary blood samples taken from fingertip shortly before and after TT was measured using Dr. Lange cuvette test LKM 140 and miniphotometer LP 20 Plus (Dr. Lange, Düsseldorf, Germany).

### 2.5. Statistical Analysis

Data were analyzed with the Statistica 13 software (TIBCO Software Inc., Palo Alto, CA, USA). The normality of data was tested using the Kolmogorov-Smirnov test. Repeated measures analysis of variance (ANOVA) was utilized to assess the differences within and between the trials. Following significant trial by time interactions, Tukeyʼs honest significant difference post hoc analysis was used for multiple comparisons. Between-trial differences in TT time and work done were analyzed using paired samples Studentʼs *t*-test. A Pearson coefficient of correlation (r) was used to determine the relationship between variables. Significance was set at *p* < 0.05 level. Data are expressed as mean ± the standard deviation of the mean (SD). The hormone and glucose concentrations measured after the TT are presented as corrected for the individual changes in PV.

## 3. Results

### 3.1. Blood Biochemical Parameters

**Figure 2:**
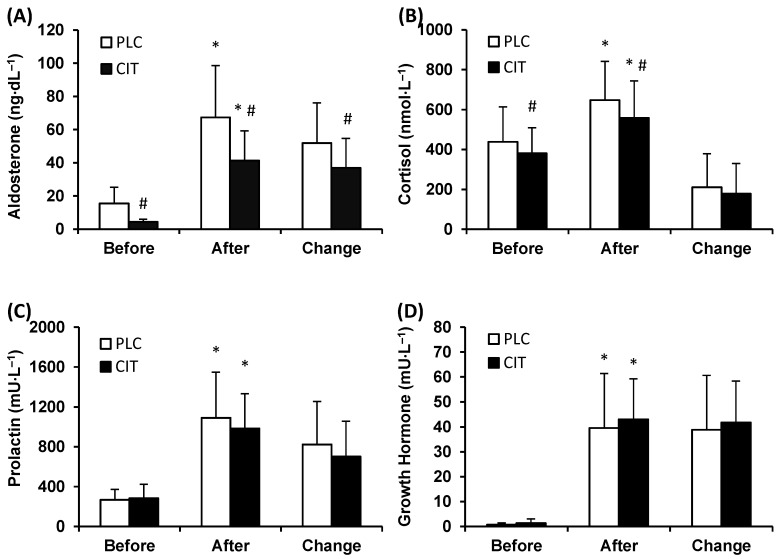
Plasma aldosterone (**A**), serum cortisol (**B**), prolactin (**C**), and growth hormone (**D**) concentration in placebo (PLC) and sodium citrate (CIT) trials before and after 40 km time-trial (mean ± SD; *n* = 20). Significantly different (*p* < 0.05): * from Before; ^#^ from PLC.

Plasma ALDO level was lower in CIT compared to PLC trial before (*p* ˂ 0.001) and after TT (*p* ˂ 0.001) (Figure 2A). During TT, ALDO levels increased in both trials, but the magnitude of the increase was smaller in CIT than in PLC trial (*p* ˂ 0.001).

Serum CORT level was lower in CIT compared to PLC trial before (*p* = 0.039) and after (*p* ˂ 0.001) TT (Figure 2B). However, the magnitude of the increase in CORT level during TT was similar in the two trials (*p* = 0.257).

The levels of serum PRL were similar in CIT and PLC trials before and after TT (*p* = 0.984 and 0.107, respectively) (Figure 2C). During TT, concentrations of PRL increased (*p* ˂ 0.001) in both trials. The between-trial difference in the extent of an increase in PRL levels did not reach statistical significance (*p* = 0.064).

Serum GH concentrations before and after TT were similar in CIT and PLC trials (*p* = 0.995 and 0.575, respectively) (Figure 2D). During TT, the levels of GH increased (*p* ˂ 0.001) in both trials to a similar extent (*p* = 0.460).

**Figure 3:**
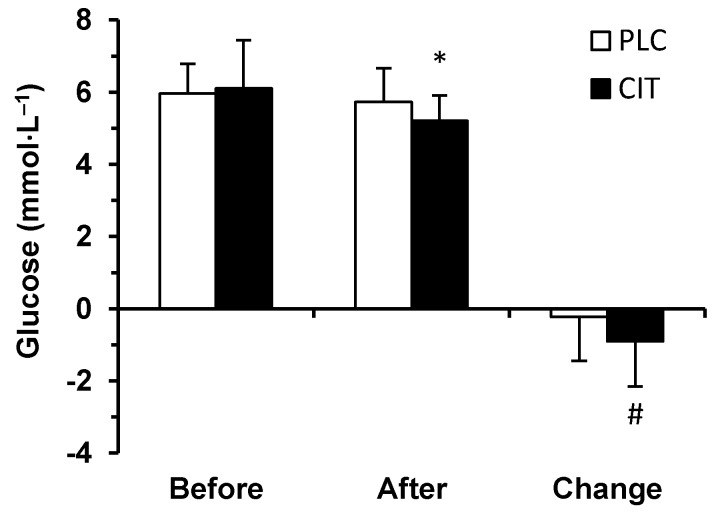
Serum glucose concentration in placebo (PLC) and sodium citrate (CIT) trials before and after 40 km time-trial (mean ± SD; *n* = 20). Significantly different (*p* < 0.05): * from Before; ^#^ from PLC.

Prior to (*p* = 0.844) and after (*p* = 0.055) TT, serum glucose concentration did not differ in the two trials (Figure 3). However, during TT, the extent of a decrease in serum glucose concentration was greater in CIT compared to PLC trial (*p* = 0.020). Therefore, after finishing the TT, serum glucose concentration had fallen below the before exercise level in CIT (*p* < 0.001) but not in PLC trial (*p* = 0.643).

Blood lactate levels before TT were similar in PLC and CIT trials (2.06 ± 0.37 and 2.31 ± 0.42 mmol·L^−1^, respectively; *p* = 0.829). However, during TT, the extent of an increase in blood lactate concentration was greater (*p* ˂ 0.001) in CIT trial compared to PLC trial. After exercise higher (*p* ˂ 0.001) blood lactate level occurred in CIT compared to PLC trial (7.58 ± 2.44 and 5.58 ± 1.32 mmol·L^−1^, respectively).

### 3.2. Time-Trial Performance, Core Body Temperature and Plasma Volume

Total work performed during TT was similar in PLC and CIT trials (864.4 ± 61.3 and 866.6 ± 68.3 kJ, respectively, *p* = 0.716), and total time also did not differ (68.11 ± 2.87 and 68.10 ± 3.28 min, respectively, *p* = 0.961). There were no between-trial differences in 5 km split times during the TT (all *p* values from 0.171–1.0). T_c_ before TT was similar in the two trials (37.09 ± 0.21 °C in CIT and 37.03 ± 0.31 °C in PLC trial, *p* = 0.185), but during exercise the magnitude of increase in T_c_ was smaller in CIT than in PLC trial (2.45 ± 0.60 °C and 2.62 ± 0.62 °C, respectively, *p* = 0.003).

Total water intake (4962 ± 739 mL in PLC and 5495 ± 721 mL in CIT) and BM gain (3.67 ± 0.75% in PLC and 4.68 ± 0.71% in CIT) during recovery period were greater (*p* < 0.001) in CIT compared to PLC trial. Compared to pre-DE time point, after DE PV had diminished by 15.7 ± 4.2% in CIT and 15.2 ± 4.6% in PLC trial (no between-trial difference, *p* = 0.422). However, at the end of the recovery period, i.e., prior to the start of TT, PV exceeded the pre-DE level by 6.9 ± 5.4% in CIT trial and remained 0.9 ± 3.3% below that in PLC trial (significant between-trial difference, *p* ˂ 0.001). After TT, PV had fallen below pre-DE level by 6.2 ± 6.3% in CIT and 12.5 ± 6.9% in PLC trial (significant between-trial difference, *p* ˂ 0.001).

### 3.3. Correlations of Hormonal Parameters with Blood Lactate Concentration, Core Body Temperature, and Serum Glucose Level

During TT, the only hormone the increases of which significantly correlated with increases in blood lactate concentration was CORT and the relationship occurred only in PLC trial: r = 0.566, *p* = 0.009. The only hormone the increases of which significantly correlated with increases in T_c_ was PRL: r = 0.609 (*p* = 0.004) in PLC and r = 0.572 (*p* = 0.008) in CIT trial. Based on pooled data from PLC and CIT trials, plasma ALDO levels before TT correlated with changes in PV during time interval between the beginning of DE and end of the recovery period (r = −0.520, *p* = 0.001). Moreover, plasma ALDO levels after TT correlated with changes in PV during time interval between the beginning of DE and end of TT (r = −0.338, *p* = 0.033). Finally, based on pooled data from PLC and CIT trials, serum CORT levels before TT correlated with serum glucose concentrations after exercise (r = 0.429, *p* = 0.006).

## 4. Discussion

This study investigates for the first time whether CIT ingestion in the amount that has been shown to induce metabolic alkalosis and an acute increase in PV [9] influences the responses of blood stress hormones to prolonged self-paced cycling exercise in the heat. We hypothesized that CIT compared to PLC would attenuate increases in the blood levels of CORT, ALDO, GH and PRL during combined exercise and heat stress. Our findings fully confirm the validity of the hypothesis only for ALDO the increase of which was significantly blunted in CIT trial during TT. Furthermore, in the CIT trial, a significantly lower level of ALDO already occurred at resting state prior to the start of the TT. Our observation of blunted ALDO response to TT together with lower ALDO concentrations before and after exercise in CIT compared to PLC trial is in good agreement with previous data that were collected in a temperate environment [10]. The novel aspect of the current study is that it demonstrates the capacity of CIT to reduce blood ALDO levels during exercise-heat stress that is strong stimulus for ALDO secretion [34]. Similarly, after CIT ingestion, significantly lower blood CORT levels occurred prior to and immediately after completing the TT. However, there was no between-trial difference in the magnitude of the increases in CORT levels that occurred during exercise. CIT ingestion did not influence GH and PRL levels in our participants.

In goats, metabolic alkalosis suppressed blood ALDO concentration in the absence of detectable changes in plasma K^+^ concentration or renin activity [38], which are considered primary regulators of ALDO secretion [39,40]. However, in sedentary rats, alkalosis did not decrease, but rather increased plasma ALDO concentration [41]. In young men, ingestion of either NaHCO_3_ or KHCO_3_ induced metabolic alkalosis, but neither of these substances suppressed plasma ALDO levels [42]. Plasma ALDO and K^+^ concentrations did not change in NaHCO_3_ trial, but in KHCO_3_ trial, parallel increases in ALDO and K^+^ levels occurred [42]. Considering these data [38,39,40,41,42], it seems unlikely that reduced ALDO levels were induced by metabolic alkalosis in our participants in CIT trial.

In temperate environmental conditions, CIT compared to PLC ingestion induced higher serum Na^+^ and lower K^+^ concentrations [10] and exactly the same pattern of changes in blood ALDO levels as observed in the current study. Therefore, it is plausible that a similar impact of CIT on blood electrolyte levels occurred in our participants. Increase in K^+^ is considered a strong, and decrease in Na^+^ a relatively weak, stimulus for ALDO secretion [39,40,43]. In light of these data [10,39,40,43], it is likely that CIT-induced changes in blood K^+^ and Na^+^ concentrations were among the factors that reduced ALDO levels in our participants in resting state and during exercise in the heat.

Similar BM, urine specific gravity and serum osmolality [35] suggest that our participants were equally hydrated and in an euvolemic status before DE in both trials. However, prior to TT, their PV had expanded by 7.8% in CIT compared to PLC trial. In temperate environmental conditions, expanded PV and decreased blood ALDO levels after CIT ingestion were observed [10]. Others [44,45] induced an acute pre-exercise PV expansion by infusion of dextran solution and reported blunted blood ALDO responses to prolonged constant-load cycling exercise. As with Grant et al. [44], we observed an inverse relationship between the PV changes and blood ALDO levels. Nevertheless, in the study by Grant et al. [44] the effect of PV expansion was manifested only during exercise, whereas in our participants decreased blood ALDO level occurred already at resting state prior to the TT. This discrepancy could be explained by the specifics of the methods used for inducing PV expansion (dextran infusion vs. CIT ingestion) and/or by different time interval during which pre-exercise PV expansion was achieved (2 h vs. 16 h). Thus, in light of previous data [10,44,45] our findings suggest that expansion of PV was one of the factors that decreased blood ALDO levels at resting state and during exercise in the heat.

Cortisol is among the most widely studied markers of stress [15,46]. In a temperate environment, NaHCO_3_ compared to PLC ingestion attenuated serum CORT responses to four consecutive 30 s bouts of maximal cycling exercise even though the mean power output was higher in NaHCO_3_ trial [22]. These authors concluded that exercise-induced acidosis stimulated CORT secretion and that NaHCO_3_ induced metabolic alkalosis was partly responsible for blunted CORT response. Without ingestion of alkalizing substances, parallel increases in blood lactate level and acidity occur during exercise [47]. Therefore, observation that during TT in PLC trial increases in serum CORT levels correlated positively with changes in lactate concentrations suggests that exercise-induced acidosis influenced CORT secretion in our participants. The absence of such correlation in CIT trial may be explained by the fact that induced metabolic alkalosis facilitates efflux of both lactate and H^+^ from contracting muscle cells and at the same time more efficiently buffers H^+^, leading to greater elevations in blood lactate levels, but attenuating increases in blood acidity [4,5,6] and CORT levels. Nevertheless, unlike Wahl et al. [22], we observed lower serum CORT level already at resting state prior to exercise in CIT trial. This discrepancy may have arisen from the fact that our participants started CIT ingestion 16 h prior to donating the pre-exercise blood sample whereas, Wahl et al. [22] administered NaHCO_3_ to their subjects only over a 90 min period preceding the start of exercise.

Cooper et al. [48] and Rhind et al. [49] reported a significant relationship between exercise-induced increases in T_c_ and plasma CORT levels. In our participants, CIT ingestion induced acute PV expansion that may slow down increases in T_c_ during exercise [11]. However, lower serum CORT level in CIT trial occurred already before the start of the TT when T_c_ did not differ. Immediately after exercise, T_c_ was lower in CIT trial, but the between-trial difference in serum CORT level was similar to that observed prior to exercise. Furthermore, increases in T_c_ did not correlate with increases in serum CORT levels during exercise in either trial. Thus, it is unlikely that T_c_ induced between-trial differences in serum CORT level in our participants.

Nutritional status, nutrient timing, intensity and duration of exercise and total amount of work completed are important factors influencing endocrine responses to exercise [16,50]. Our participants’ energy and nutrient intake and timing was carefully controlled and kept identical in PLC and CIT trials during the 16 h recovery period [35]. Time-trial time and total amount of work completed during exercise did not differ in the two trials. Thus, these factors could not induce the between-trial differences observed in ALDO and CORT levels. Similarly, these factors do not explain greater decrease in serum glucose concentration in CIT compared to PLC trial during exercise. On the other hand, CORT possesses a crucial role in maintaining blood glucose level [16], and it has been shown that during prolonged exercise, lower CORT levels are associated with greater decreases in plasma glucose concentrations [51]. In light of these data [16,51] and considering positive correlation observed between pre-exercise CORT and post-exercise glucose concentrations in our participants, it is plausible that during TT in CIT trial, the greater decrease in serum glucose concentration was at least partly due to lower CORT level.

Several-fold increases in serum PRL concentrations occurred during TT, but the magnitude of this response was similar in the two trials. Exercise stimulates PRL secretion [52], whereas the magnitude of rise in blood PRL level in response to similar exercise demands is markedly greater in the heat compared to temperate [28,53] or cold [54,55] environments. De Meirleir et al. [56] suggested that the trigger mechanism of PRL release is exercise-induced metabolic acidosis, and Rojas Vega et al. [24] found that metabolic alkalosis reduced PRL response to short-duration exhausting exercise in a temperate environment. Considering the proven alkalotic effect of the dose used and protocol of administration of CIT [9], our findings suggest that orally induced metabolic alkalosis did not affect serum PRL response to TT in the heat. Several factors may explain the discrepancy between our data and those of Rojas Vega et al. [24], including differences in environmental conditions and exercise demands. Luger et al. [57] and Schulte et al. [58] reported that metabolic alkalosis induced by sodium lactate infusion did not decrease but increased blood PRL levels in sedentary subjects. As blood lactate concentration rose to the levels that usually occur during high-intensity work, Luger et al. [57] concluded that lactate per se, i.e., independently of acidosis, may trigger PRL release. In our participants, no relationship between exercise-induced changes in blood lactate and PRL concentrations occurred.

A significant relationship occurs between T_c_ and PRL response to exercise [28,59,60,61]. Our data are consistent with this observation as exercise-induced increases in serum PRL concentrations correlated with increases in T_c_. Taken together, our data suggest that increases in T_c_ stimulate PRL secretion during prolonged cycling exercise in the heat and that CIT ingestion does not modulate serum PRL response in this situation.

Secretory stimuli are similar for PRL and GH [24]. Similar general patterns of GH and PRL serum levels in our participants, including the absence of any effect of CIT ingestion, are in accordance with this notion. However, Gordon et al. [23] and Wahl et al. [22] reported that metabolic alkalosis attenuated blood GH response to short-term high-intensity exercise in temperate environmental conditions. Assuming that our participants were in an alkalotic state in CIT trial, our data suggest that alkalosis did not modulate serum GH responses to prolonged exercise in the heat. On the other hand, Luger et al. [57] observed increased but not decreased blood GH levels in sedentary subjects after inducing metabolic alkalosis by infusion of sodium lactate and concluded that, independently of acidosis, lactate may stimulate GH secretion also during exercise. In our participants, no relationship between exercise-induced changes in blood lactate and GH levels occurred.

During the TT serum GH level increased by several times more than that of PRL and, differently from PRL, changes in GH level did not correlate with increases in T_c_. Literature shows, with a few exceptions [59,62], that GH secretion is sensitive to increases in T_c_ [30,55,63], and that positive relationships occur between T_c_ and GH levels during prolonged exercise in the cold [64] and in the heat [31,49,65]. However, all previous studies which revealed direct relationship between exercise-induced changes in T_c_ and blood GH levels employed constant-load exercise of fixed duration [49,64,65] or to volitional exhaustion [31], whereas in the current study, the participants performed self-paced TT. Thus, the mode of the exercise protocol, i.e., self-pacing, might be a factor confounding the relationship between changes in T_c_ and serum GH in our participants. Nevertheless, our data reveal that in the heat, prolonged self-paced cycling exercise induces immense increases in serum GH levels, and that CIT ingestion does not modulate GH response in these conditions.

Altogether, our findings of reduced ALDO and CORT levels before and after TT and blunted acute response of ALDO to TT suggest that CIT ingestion after DE alleviates stress during subsequent heavy endurance cycling bout in a warm environment. As suggested by Peart et al. [18], reduced stress during exercise may lead to improved recovery. This possibility deserves further research, because in multi-stage road cycling races, improved recovery between stages may strongly influence the final rankings of the competitors. Therefore, investigating only one exercise-recovery-exercise cycle may be considered an important limitation of this study. Another important limitation is the absence of data regarding the levels of hormones and glucose pre- and post-DE. Limitations of our study also include lack of definite data characterizing blood acid-base balance and electrolyte levels. Nevertheless, in a previous study [9], the CIT supplementation strategy used in the current investigation resulted in significant increases in blood pH, bicarbonate ion concentration and base excess. Also, CIT ingestion has been shown to increase serum Na^+^ and decrease K^+^ concentrations [10].

## 5. Conclusions

CIT compared to PLC ingestion during 16 h recovery period after DE results in decreased blood ALDO and CORT levels prior to and during subsequent cycling TT in a warm environment in male non-heat-acclimated endurance athletes. CIT ingestion also reduces the magnitude of acute increases in blood ALDO, but not CORT level, during TT. CIT ingestion has no influence on blood levels of PRL or GH in these experimental conditions.
